# Acute gastroesophageal intussusception in a juvenile Australian shepherd dog: endoscopic treatment and long-term follow-up

**DOI:** 10.1186/1746-6148-10-109

**Published:** 2014-05-07

**Authors:** Sarina Shibly, Sandy Karl, Katharina M Hittmair, Reinhard A Hirt

**Affiliations:** 1 Department for Small Animals and Horses, Clinic of Internal Medicine and Infectious Diseases, University of Veterinary Medicine Vienna, Veterinaerplatz 1, Vienna A-1210, Austria; 2 Department for Small Animals and Horses, Clinical Section of Diagnostic Imaging, University of Veterinary Medicine Vienna, Veterinaerplatz 1, Vienna A-1210, Austria

**Keywords:** Esophageal obstruction, Canine, Eso2phagoscopy, PEG tube, Gastroesophageal intussusception

## Abstract

**Background:**

Canine gastroesophageal intussusception (GEI) is a rare and potentially fatal disease usually affecting puppies or young dogs < 3 months of age and of medium to large breeds. Surgical intervention has been advocated as the therapy of choice by most authors. Endoscopic treatment may offer an advantageous or alternative method of treatment.

**Case presentation:**

GEI was diagnosed in a nine-week-old Australian Shepherd dog with an acute onset of vomiting and regurgitation and compatible radiographic findings on thoracic radiography. Treatment consisted of endoscopic gastric repositioning and placement of a percutaneous endoscopic gastrostomy (PEG) tube to prevent repeated dislocation of the stomach, and to allow for nutritional supplementation During a follow- up period of eight months, thoracic radiographs were obtained showing persistent esophageal dilatation in the absence of compatible clinical signs.

**Conclusion:**

Endoscopic intervention is an effective, alternative in selected canine GEI- cases, allowing for rapid, minimally invasive confirmation of diagnosis and therapy. After initial treatment, radiographic long-term follow-up seems prudent even in asymptomatic patients.

## Background

In dogs, esophageal dysfunction is usually not difficult to diagnose, as patients show typical clinical signs such as regurgitation, dysphagia, odynophagia, repeated swallowing attempts and excessive salivation. However, identification of a definitive diagnosis of esophageal disease which allows specific therapy can be challenging and require laboratory testing, radiography and endoscopy [1,2].

GEI is a condition in which the stomach (in total or in part) is translocated into the intrathoracic esophageal lumen [3-7]. Additionally, adjacent organs (e.g. duodenum, spleen, pancreas, omentum) may also invaginate [3-5]. In contrast to GEI, hiatal hernias are characterized either by cranial malpositioning of the gastroesophageal junction (sliding hernia) or by cranial displacement of the stomach adjacent to the esophagus [8,9].

Canine GEI is a rare disease usually affecting puppies of medium to large breeds younger than three months of age [3]. Males and German Shepherd dogs seem to be overrepresented, the latter possibly because of their predisposition for congenital megaesophagus [4-6]. The patient reported here is a female and belongs to a breed in which GEI, to the authors’ knowledge, has not been previously described.

## Case presentation

A nine-week-old, female Australian Shepherd dog weighing five kg was presented to the Emergency Department of the University of Veterinary Medicine Vienna, Austria, with a three-day history of profuse vomiting and regurgitation. No clinical signs had been noticed before the acute onset of disease. The puppy had been purchased from a breeder one week prior to presentation. The primary veterinarian suspected a hiatal hernia based on thoracic and abdominal positive contrast radiographs. The puppy had been fed solid food for a couple of weeks without any difficulty, had been healthy from birth, and was the largest puppy of the litter.

Initial physical examination revealed lethargy, a body condition score of four out of nine, reduced skin turgor, increased vesicular lung sounds and abdominal tenderness on palpation. Other parameters were within normal limits. A fecal test for parvoviral antigen (IDEXX SNAP® test) was negative.

Clinicopathologic findings on blood examination were metabolic alkalosis (pH 7.51 [reference value 7.351-7.463], HCO3 38.6 mmol/L [reference value 18-24 mmol/L]), hypokalemia (2.9 mmol/L [reference value 3.6-5.6 mmol/L]), both likely attributable to the vomiting, and a mild, stress- induced hyperglycemia (132 mg/dl [reference value 55-100 mg/dl]).

**Figure 1:**
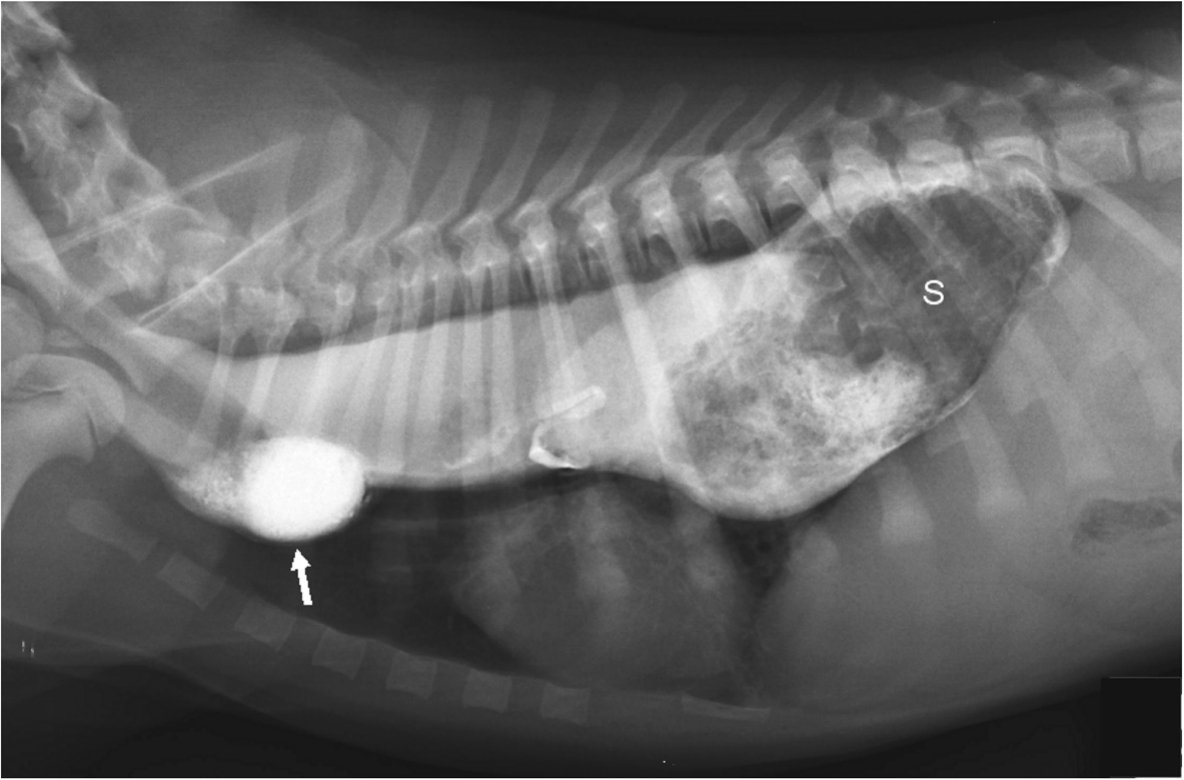
**Thoracic radiograph in left lateral recumbency of the dog on the day of presentation.** The esophagus is filled with barium contrast and is dilated. The stomach (S) is invaginated into the esophagus. There is a suspected diverticulum of the esophagus cranial to the heart (arrow).

Thoracic radiographs in left lateral recumbency showed a contrast-filled esophagus, due to a barium contrast study performed by the referring veterinarian 4 hours previously. A diverticulum of the esophagus in the cranial mediastinum was suspected with severe esophageal distention in the caudal portion of the mediastinum. An intraluminal soft-tissue mass with traces of barium contrast and rugal folds was visible in the caudal thorax. The trachea was displaced ventrally, and the stomach silhouette was not visible in the cranial abdomen. There was no evidence of lung consolidation or infiltration, although esophageal obstruction caused by gastroesophageal intussusception (GEI) was strongly suspected (Figure 1).

Initial treatment for dehydration, and electrolyte imbalances from the regurgitation and vomiting consisted of intravenous saline supplemented with potassium chloride (6 ml/kg/h) to balance dehydration and hypokalemia, ranitidine (2 mg/kg IV BID, Ulsal Injectable, Gebro Pharma GmbH, Fieberbrunn, A) as gastric protectant, maropitant (1 mg/kg IV SID, Cerenia Injectable, Pfizer Animal Health Austria GmbH, Vienna, A) as antiemetic, and amoxicillin-clavulanic acid (22 mg/kg IV BID, Clavamox Injectable, 550 mg, Sandoz GmbH, Vienna, A) to prevent possible aspiration pneumonia. Esophageal endoscopy was performed under general anesthesia (butorphanol 0.1 mg/kg IV, Butomidor Injectable, 10 mg/ml, Richter Pharma AG, Wels, A; propofol 5 mg/kg IV, Propofol „Fresenius“ 1% with MCT Injectable, Fresenius Kabi Austria GmbH, Graz, A; and inhalational isoflurane).

**Figure 2:**
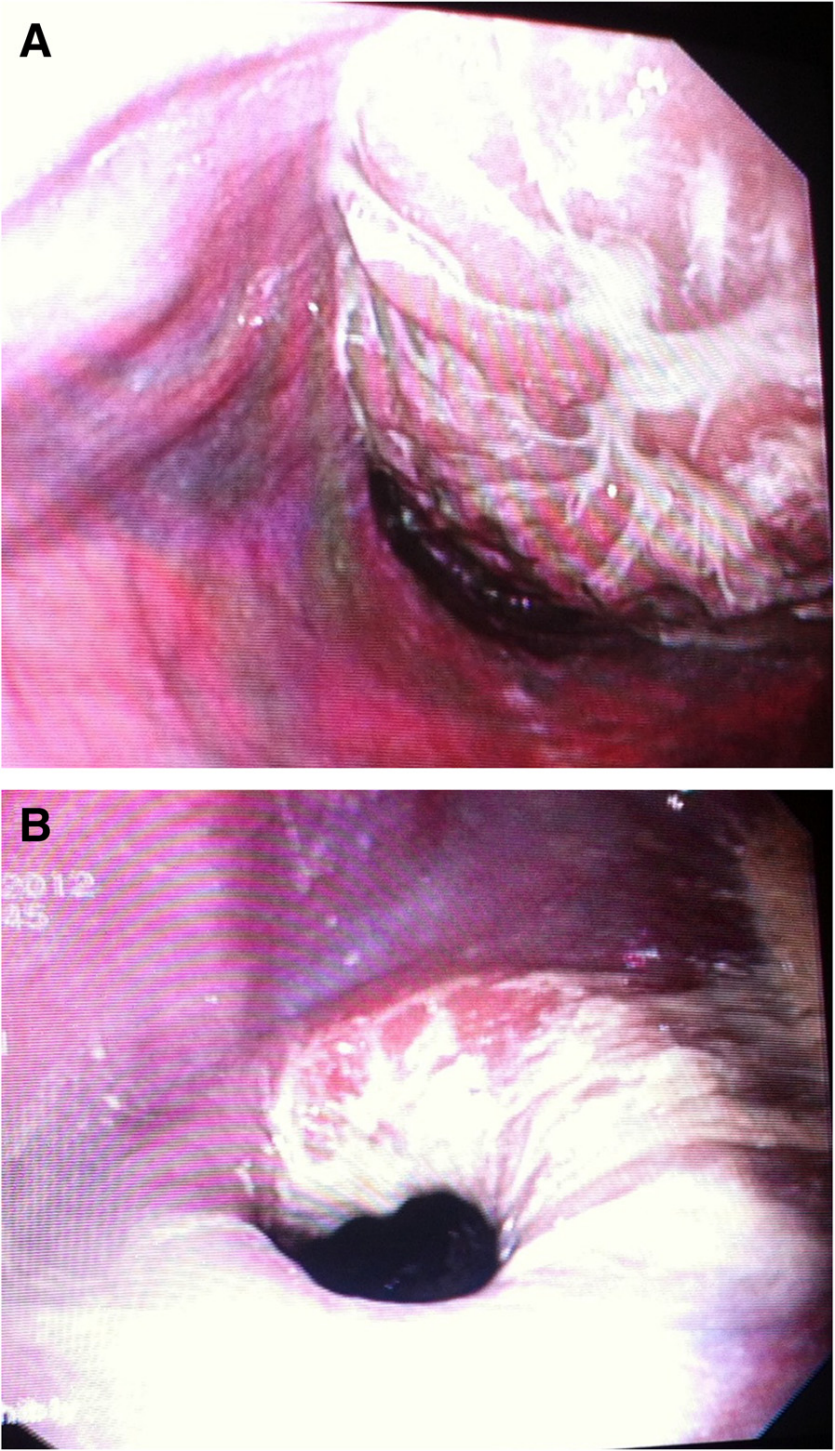
**Esophageal lumen. (A)** Obstruction by intussuscepted stomach. **(B)** Incomplete cardiac closure following gastric Reposition.

Inspection of the esophagus with a flexible videoendoscope (Olympus GIF 165) revealed accumulation of intraluminal fluid, food particles and contrast media in the cranial and midsection of the esophagus, while the caudal third of the esophagus appeared distended and obstructed by an intraluminal mass consistent with the stomach (Figure 2A). Uncomplicated gastric reposition was achieved by advancement of the endoscope against the gastric mucosa. Closure of the gastric cardia appeared incomplete. The caudal esophagus remained dilated with the mucosa macroscopically intact (Figure 2A). To prevent repeated dislocation of the stomach and to allow for nutrition bypassing the esophagus, a percutaneous endoscopic gastrostomy (PEG) tube (mushroom/Pezzar style silicone catheter, Surgivet, Smiths Medical, Dublin, OH, USA) was placed in the left abdominal wall. Inspection of the cranial and middle parts of the esophageal lumen after removal of its contents failed to demonstrate the presence of the radiographically suspected cranial diverticulum. Based on endoscopic findings the final diagnosis was GEI with secondary esophageal dilation.

After an uneventful recovery from anesthesia, the patient’s condition improved. Partial parenteral nutrition, a metoclopramide constant rate infusion (CRI, 0.01 mg/kg/h, Paspertin 10 mg Injectable, Abbott Products GmbH, Hannover, D) to prevent vomiting and a lidocaine CRI (0.05 mg/kg/h, Xylanaest purum 1% Injectable, Gebro Pharma GmbH, Fieberbrunn, A) as analgetic and radical scavenger were added to the therapeutic regimen. Twelve hours after endoscopy, lidocaine was progressively reduced and discontinued. A mucosal protectant (sucralfate, 0.1 g/kg PO TID, Ulcogant oral suspension 1 g/5 ml, Merck S.L., Mollet Del Valles, E) was administered, along with small amounts of water. Feeding via the PEG tube was withheld for another 24 hours to prevent possible mucosal irritation caused by gastroesophageal reflux or vomiting.

**Figure 3:**
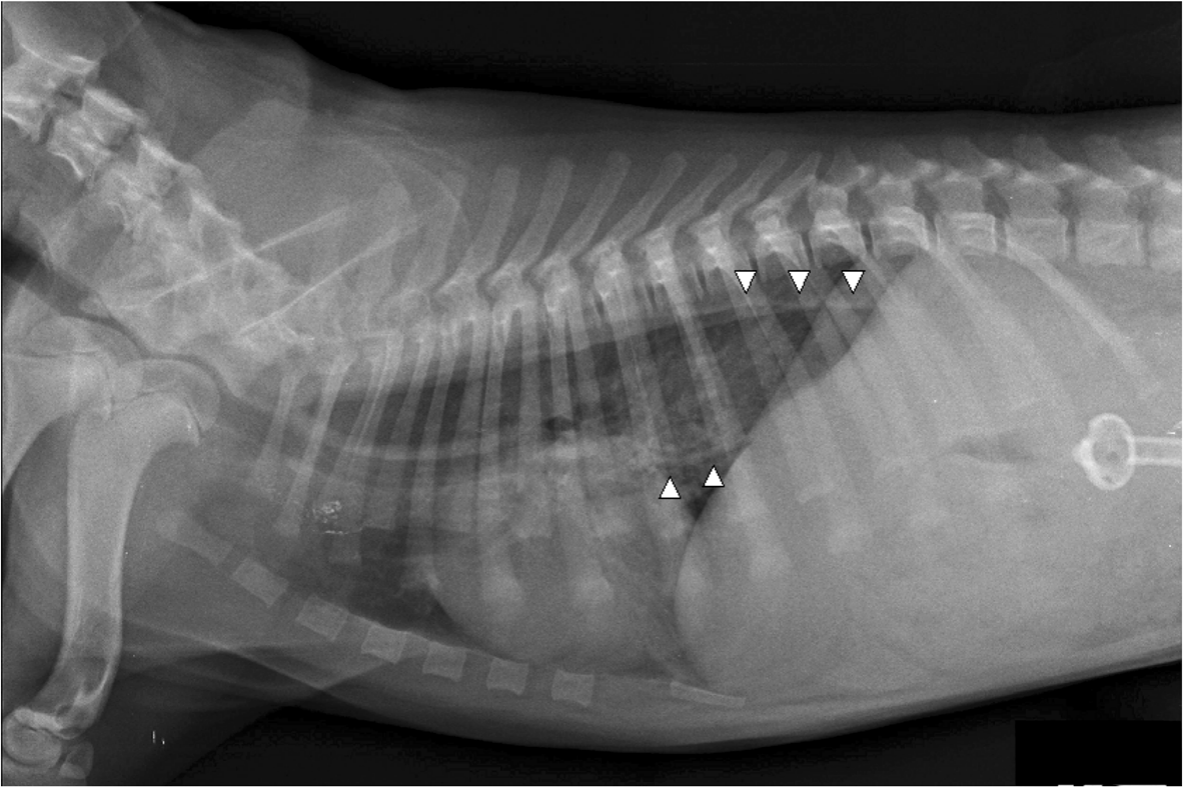
**Thoracic radiograph in left lateral recumbency 36 hours after gastric repositioning.** The esophagus is dilated (arrowheads) and there are no traces of contrast agent left. A mild interstitial and alveolar lung pattern indicates aspiration pneumonia. A PEG tube is visible in the abdomen.

Thoracic radiographs in left lateral recumbency taken 36 hours after gastric repositioning showed resolution of the gastroesophageal intussusception. The esophagus still appeared dilated, and an interstitial and mild alveolar lung pattern was identified and thought to be from aspiration pneumonia (Figure 3).

Thirty-six hours after endoscopy, enteral nutrition was initiated via PEG tube using a bland diet. The dog was clinically normal, playful, tolerated oral water and sucralfate well. Over the next days, blood values returned to normal and oral feeding from an elevated position was gradually introduced. Although neither vomiting nor regurgitation were observed, thoracic radiographs in left lateral recumbency nine days after initial presentation showed persisting caudal esophageal dilation.

The patient was discharged nine days after admission. The owner was instructed to offer incrementally increasing small portions of food formed to meatballs from an elevated position five times daily and was taught how to use the PEG tube to maintain the dog’s nutrition. Ranitidine, sucralfate and amoxicillin-clavulanic acid were prescribed as oral medications. The dog remained clinically unremarkable, gained weight and size quickly, and the PEG tube was removed two weeks after discharge as oral feedings covered the patient’s nutritional demands.

**Figure 4:**
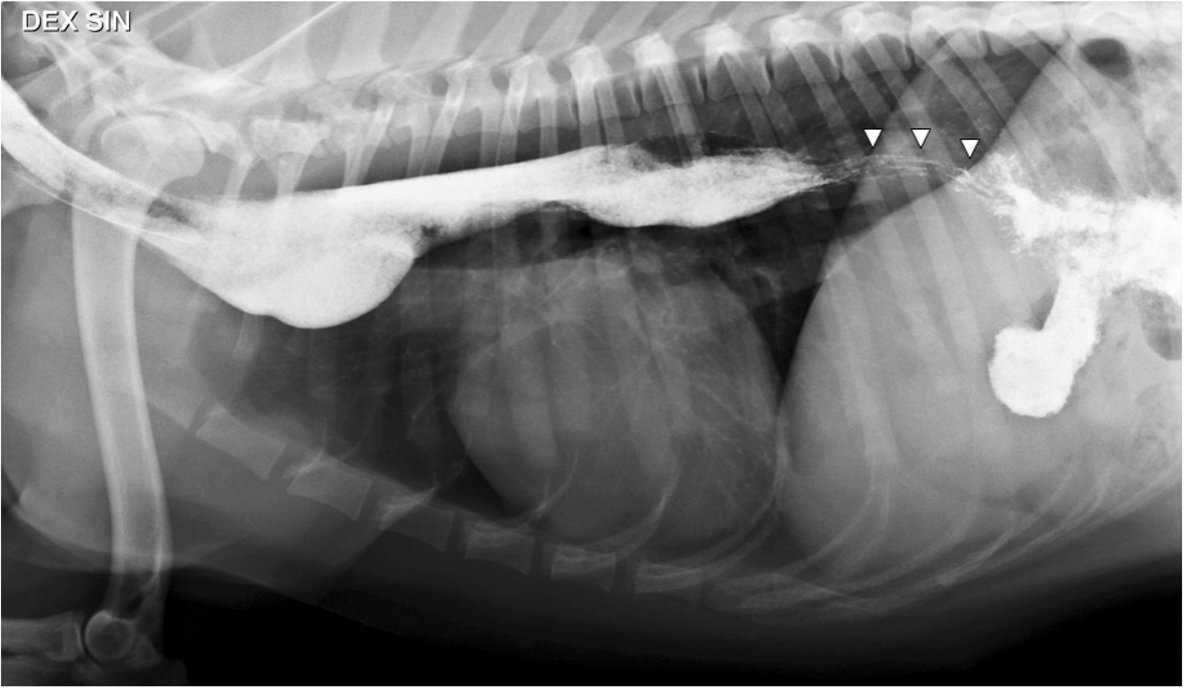
**Thoracic radiograph with esophageal contrast study in left lateral recumbency eight months after diagnosis.** The esophagus is dilated and filled with barium contrast and there is a slight distension or diverticulum is located cranial to the heart. There is a striped pattern in the caudal esophagus, and the stomach is filled with contrast agent.

Thoracic radiographs in left lateral recumbency repeated at five weeks, four months and eight months after discharge revealed persistent esophageal dilatation, absence of abnormal lung patterns, and the patient continues to tolerate commercially available dog food and treats without difficulty. Thoracic radiographs in left lateral recumbency eight months after initial presentation show the esophagus is still dilated with a slight distension cranial to the heart. There is a striped luminal pattern in the caudal section consistent with a narrower lumen of the esophagus. The stomach is filled with contrast medium (Figure 4).

## Discussion

GEI has been reported in different species, including adult and pediatric humans [10,11], dogs [3,7,12-19], domestic cats [4,20,21] and an adult leopard [22]. While the chronic recurrent form causes intermittent gastrointestinal signs, patients with acute persistent GEI present with an acute onset of clinical signs from esophageal obstruction, occasionally accompanied by respiratory distress caused by the intrathoracic mass effect and/or aspiration pneumonia [19,23]. Additional clinical signs of acute disease include regurgitation, vomiting, drooling, dysphagia and abdominal discomfort [8,13]. Reduced venous return, endotoxic shock and release of inflammatory mediators can lead to cardiovascular impairment and rapid death [17]. The case reported here can be classified as acute GEI, as no abnormalities had been noticed until three days prior to presentation.

GEI occurs with reverse gastric peristalsis in combination with a sudden sustained increase in abdominal pressure [3]. The pathogenesis of this condition has not been fully elucidated and is likely multifactorial [6]. Abnormalities including esophageal motility disorders, hiatal enlargement and lower esophageal sphincter failure presumably predispose to GEI [8,9].

In human medicine, adults are affected more often than children, and risk factors include eating disorders, alcohol abuse, sudden sustained exertion, small-bowel obstruction, acid bile peptic disease and pregnancy [10,11]. In cats, chronic intermittent GEI predominates [4,20,21], while dogs tend to develop the acute form of the condition [3,12,14,17,18,23]. In dogs, formation of GEI has been linked to increased intraabdominal pressure from vomiting or blunt trauma [6], negative intrathoracic pressure caused by respiratory disease [6,24] as well as preexisting esophageal disease, especially megaesophagus [3,6-8].

Canine GEI usually affects puppies of medium and large breeds younger than three months of age [3] with males and German Shepherd dogs being overrepresented [4-6]. The patient reported here is a female and belongs to a breed in which GEI, to the authors’ knowledge, has not been described before.

It may be speculated that the patient described in this report developed GEI as a result of an acute vomiting episode (e.g. caused by stress due to relocation or possible dietary changes). This is supported by breeder’s account that neither regurgitation nor dysphagia had been observed before the acute onset of signs at nine weeks of age. Furthermore, the puppy reportedly had been the largest of the litter, which is an unexpected historical finding in a dog with congenital esophageal disease. On the other hand, radiographic evidence of esophageal dilatation which was not identified during endoscopy persisted over months following gastric repositioning without causing concurrent clinical signs. This finding argues against a reversible esophageal dilatation caused by the intussuscepted stomach, and demonstrates that disappearance of clinical signs does not necessarily equal spontaneous resolution of congenital esophageal disease [7]. Probably a combination of a congenital esophageal disorder combined with severe vomiting due to other causes triggered GEI in the case described here.

Procedures to diagnose GEI include survey or contrast radiography, fluoroscopy and endoscopy [4,7]. In the present case, contrast radiography revealed an intraluminal soft tissue mass within the caudal thoracic esophageal lumen with vertical lamellar accumulations of contrast medium compatible with gastric folds. The definitive diagnosis of GEI was established using esophagoscopy.

Dogs that suffer from GEI can deteriorate rapidly with mortality rates up to 95% reported for complicated cases [4,5]. Patient stabilization and repositioning of the translocated viscera needs to be initiated when there are acute presentations with little delay [3].

Generally, surgical intervention with reduction of the intussusception followed by gastropexy has been advocated as the treatment of choice by most authors [5,14,15,17,18,23]. Potential disadvantages include prolonged general anesthesia [7] in a potentially hemodynamically compromised, sometimes malnourished patient, the invasiveness and expense of surgery, and the inability to fully evaluate the condition of the esophageal and gastric mucosa, which may impact prognosis. Furthermore, an extended convalescence period, and possible postsurgical complications such as wound infections or delayed tissue healing need to be considered. These factors, in addition to potential congenital esophageal abnormalities resulting in a guarded prognosis [7], may influence decision-making leading to euthanasia of a patient with a potentially controllable condition.

Flexible endoscopy is a minimally invasive technique requiring a brief general anesthestic procedure and offering a number of diagnostic and therapeutic options. In the management of GEI, endoscopy allows for definitive diagnosis of the condition, evaluation of the integrity of the upper gastrointestinal tract lining, repositioning of the prolapsed stomach as well as placement of a PEG tube serving as gastropexy-device and enabling enteral nutrition bypassing a possibly impaired esophagus [7]. One limitation of the endoscopic approach is the inability to assess and correct concurrent problems such as hiatal abnormalities. Furthermore, with PEG tube placement only a unilateral gastropexy is established, whereas bilateral gastropexy has been recommended by some authors for management of GEI [15,17]. Interestingly, left unilateral gastropexy has been advocated by other authors, since this technique results in positioning of the esophageal hiatus to the left of the midline possibly improving the therapeutic success rate [14,23]. Endoscopic repositioning of the stomach needs to be performed with caution and should not be attempted in cases of adhesions or evident compromise of the gastric wall to avoid iatrogenic perforations [7]. Furthermore, the procedure should be carried out by an experienced clinician to minimize complications and duration of anesthesia.

To the authors’ knowledge, three cases of endoscopic reductions of GEI have been reported. One in a cat and the other two cases in dogs [7,8,20]. The patient described by McGill and coworkers [7] was a seven-week-old female Siberian husky with esophageal dilatation and GEI and the only case to date using PEG tube placement for definitive treatment. The nine-week-old female Australian Shepherd described here had an unremarkable history up to the acute vomiting episode that had started three days prior to presentation. After radiographic evaluation and initial medication, flexible endoscopy allowed for verification of diagnosis and successful treatment of GEI within only three hours after admission leading to a rapid clinical recovery. Long-term follow-up including consecutive thoracic radiographs revealed persisting esophageal dilatation despite complete absence of compatible clinical signs, indicating that repeated diagnostic imaging might be reasonable to enable continued patient care and owner education, as clinical presentation may not reflect esophageal abnormalities present necessitating particular attentiveness.

## Conclusion

Acute GEI is a rare and potentially fatal disease and should be considered as a differential diagnosis in patients with acute signs of esophageal obstruction in all dogs. Endoscopic treatment of GEI consisting of gastric repositioning and PEG tube placement appears to be an effective, cost-efficient alternative to conventional surgery.

## Abbreviations

CRI: Constant rate infusion; GEI: Gastroesophageal intussusception; PEG tube: Percutaneous endoscopic gastrostomy tube.

## Competing interests

None of the authors has financial or personal relationships that could inappropriately influence or bias the content of the paper.

## Authors’ contributions

SS and SK were responsible for the study design. SK was responsible for initial treatment of the dog. Endoscopic intervention was performed by SS. KMH was responsible for diagnostic imaging. RAH, SK and SS were responsible for clinical examinations and medical care of the patient. SS and SK drafted the manuscript. SS, SK, KMH and RAH were involved in work supervision and writing of the manuscript. All authors read and approved the final manuscript.
